# The Severe Adverse Reaction to Vitamin K_1_ Injection Is Anaphylactoid Reaction but Not Anaphylaxis

**DOI:** 10.1371/journal.pone.0090199

**Published:** 2014-03-04

**Authors:** Yan-Ni Mi, Na-Na Ping, Xue Xiao, Yan-Bing Zhu, Jing Liu, Yong-Xiao Cao

**Affiliations:** Department of Pharmacology, Xi'an Jiaotong University College of Medicine, Xi'an, Shaanxi, China; McGill University, Canada

## Abstract

The severe adverse reaction to vitamin K_1_ injection is always remarkable and is thought to result from anaphylaxis. Paradoxically, however, some patients administered vitamin K_1_ injection for the first time have adverse reactions. Using beagle dogs, the present study tested the hypothesis that the response to vitamin K_1_ is an anaphylactoid reaction. The results showed that serious anaphylaxis-like symptoms appeared in beagle dogs after the administration of vitamin K_1_ injection for the first time. The plasma histamine concentration increased, and blood pressure decreased sharply. After sensitization, dogs were challenged with vitamin K_1_ injection and displayed the same degree of symptoms as prior to sensitization. However, when the vitamin K_1_ injection-sensitized dogs were challenged with a vitamin K_1_-fat emulsion without solubilizers such asTween-80, the abnormal reactions did not occur. Furthermore, there was no significant change in the plasma immunoglobulin E concentration after vitamin K_1_ challenge. Following treatment with vitamin K_1_ injection, the release of histamine and β-hexosaminidase by rat basophilic leukemia-2H3 cells as well as the rate of apoptosis increased. The Tween-80 group displayed results similar to those observed following vitamin K_1_ injection in vivo. However, the dogs in the vitamin K_1_-fat emulsion group did not display any abnormal behavior or significant change in plasma histamine. Additionally, degranulation and apoptosis did not occur in rat basophilic leukemia-2H3 cells. Our results indicate that the adverse reaction induced by vitamin K_1_ injection is an anaphylactoid reaction, not anaphylaxis. Vitamin K_1_ injection induces the release of inflammatory factors via a non-IgE-mediated immune pathway, for which the trigger may be the solubilizer.

## Introduction

Vitamin K (VK) is an essential factor that is required for the post-translational modification of coagulation factors II, VII, IX and X, protein C and protein S (the natural inhibitors of coagulation). VK occurs naturally in two forms, VK_1_ and VK_2_. Since the synthesis of the first synthetically prepared fat-soluble VK_1_ in 1953 [1], VK_1_ injection has been the most commonly employed preparation of a coumarin antagonist and widely used in the treatment of hemorrhagic disease caused by VK_1_ deficiency. Soon after its introduction, reports of severe adverse reactions began to appear, and the number of adverse reactions to VK_1_ injection is always remarkable. The consistent symptoms include facial flushing, vague uneasy feelings of weakness, abdominal and low back pain, nausea, vomiting, dyspnea, and chest pain. In severe reactions, these symptoms are followed within minutes by cyanosis, loss of consciousness, and hypotension, with the potential for cardio-pulmonary arrest and death [2], [3].

The adverse reaction to VK_1_ injection is believed by many to be classified as anaphylaxis. In December 2011, the State Food and Drug Administration, China, and the National Center for Adverse Drug Reaction Monitoring, China, issued a notice raising concern regarding serious anaphylaxis resulting from VK_1_ injection [4]. The database in the State Food and Drug Administration contained a total of 8146 cases of adverse reactions between January 2004 and May 2011. The adverse reactions were primarily associated with the intravenous administration of VK_1_ (95.3%). The United States Pharmacopeia and the Martindale Extra Pharmacopeia have reported that VK_1_ can cause hypersensitization. The medical use instructions for VK_1_ products from Merck, US, report that VK_1_ can induce anaphylaxis. After reviewing the reaction description (CONSTART terms, “Coding symbols for a thesaurus of standard adverse reaction terms”) in the Food and Drug Administration SRSAR file, Louis found that the patients who experienced such a reaction were never identified as “anaphylactoid” [2].

Although most of these episodes have been described as anaphylaxis in studies of clinical cases [2], [5]–[8], the number of these reactions that were IgE-mediated is unclear. Furthermore, Yang [9] collected 46 cases of allergicshock for vitamin K_1_ injection, and 19 patients (42%) were administered vitamin K_1_ injection for the first time. These results are contradictory to the mechanism of anaphylaxis. Some reports have classified these adverse reactions as anaphylactoid reactions [10]. Riegert-Johnson has reported one case of an adverse reaction to VK_1_ and concluded that this patient most likely experienced an anaphylactoid (non-IgE-mediated) hypersensitivity reaction [11]. Fiore has defined “anaphylactoid” as an adverse drug event in which at least one of the reported adverse drug reactions includes any of the following CONSTART entries: anaphylaxis, allergic reaction, apnea, death, cardiac arrest, hypotension, shock or vasodilation [2]. The attempt to identify the nature of VK_1_ injection-induced adverse reactions as anaphylaxis or anaphylactoid reactions has clearly been relatively unsuccessful. Paradoxically, VK_1_ is required in the body to participate in coagulation but can also cause anaphylaxis. On the other hand, Tween-80, a non-ionic surfactant, is the most extensively used solubilizer in lipid-soluble drugs containing VK_1_. Growing evidence suggests that Tween-80 used as a solubilizer can induce anaphylactoid reactions [12]–[14]. Therefore, we considered whether VK_1_ injection-induced adverse reactions are anaphylactoid reactions triggered by the solubilizer. The objectives of the present study were to ascertain the nature of the adverse reactions to VK_1_ injection. Clearly distinguishing the types of adverse reactions and determining the trigger will be significant in finding solutions to prevent and reduce the adverse reactions.

## Materials and Methods

### Reagents

The following reagents were used in this study: VK_1_ injection (Cisen Pharmaceutical Co., Shandong, China,), VK_1_-fat emulsion (VK_1_-FE) (Anjian Pharmaceutical Co., Xi'an, China), Tween-80 (Sigma Co., UK), adrenaline hydrochloride injection (Shanghai Harvest Pharmaceutical Co., Ltd, Shanghai, China), diphenhydramine hydrochloride injection (Tianjin Jinyao Amino Acid Co., Ltd, Tianjin, China), p-nitrophenyl-N-acetyl-β-D- glucosaminide (Sigma Co., UK), IgE ELISA kits (KeyGENBioTECH, Nanjing, China), histamine ELISA kits (KeyGENBioTECH, Nanjing, China), and Annexin V-FITC apoptosis detection kits (KeyGENBioTECH, Nanjing, China).

### Ethics Statement

This study was carried out in strict accordance with the recommendations in the Guide for the Care and Use of Laboratory Animals of the National Institutes of Health. The experimental protocols for using beagle dogs were approved by the Animal Ethics Committee at Xi'an Jiaotong University, Xi'an, China(Permit Number: XJTU 2011-0045). All surgeries were performed under sodium pentobarbital anesthesia. Adrenaline hydrochloride injection and diphenhydramine hydrochloride injection were available and ready for use to ensure dogs safety.

### Animals and cells

Beagle dogs were purchased from Xi'an Dilepu Biology Resources Development Co., Ltd. (Xi'an, China). Forty-two healthy beagle dogs (twenty females and twenty-two males), aged 2 to 3 years and with a body weight ranging from 8 to 10 kg, were included in the study. The dogs were housed in individual cages in a large colony room, with free access to water, and were fed a standard dry food twice a day. Breeding environment: The room temperature was 20∼25°C, with a relative humidity of 40%∼70% and a day-night cycle of 12/12 h. The dogs were randomly divided into 7 groups as following (6 dogs in each group): control, ovalbumin, VK_1_ injection (0.25 and 0.085 mg/kg), VK_1_-FE (0.25 and 1 mg/kg), and Tween-80 (1 mg/kg). The dogs were anesthetized by the intravenous injection of pentobarbital sodium (30 mg/kg) and immobilized on the operating table while the blood pressure was measured via a BL-420 biological function system by the femoral artery intubation. All animals were breathing spontaneously during surgery.

RBL-2H3 mast cells were purchased from the Chinese Academy of Sciences Cell Bank (Shanghai, China). The cells were cultured in a humidified atmosphere containing 5% CO_2_ at 37°C using Modified Eagle's Medium supplemented with 10% heat-inactivated fetal bovine serum, penicillin (80 units/ml) and streptomycin (0.08 mg/ml). The cells were harvested, resuspended at a concentration of 4×10^5^/ml in Modified Eagle's Medium, and plated in 24-well flat-bottomed tissue culture plates. The following day, the cells were washed with phosphate buffer solution. The cells were treated with different concentrations of VK_1_ (0.2, 2, 20, and 200 µg/ml), Tween-80 (1, 10, 100, and 1000 µg/ml), VK_1_-FE (0.2, 2, 20, and 200 µg/ml), 0.1% Triton, or Modified Eagle's Medium as a control, and incubated for 30 min at 37°C.

### Behavioral research

In the anaphylactoid reaction experiments, the drugs were intravenously administered to the dogs via a micro-injection pump at 0.4 ml/min. The behaviors of the dogs were observed and recorded for 30 min. In the anaphylaxis experiments, the dogs were sensitized with intravenous drugs every other day for a total of three administrations. On the 10th day after the last sensitization, the dogs were challenged intravenously, and their behaviors were observed. The challenge doses were twice the doses used for sensitization. A cross-challenge experiment was implemented, in which the dogs sensitized with VK_1_ injection were challenged with VK_1_-FE or the dogs sensitized with VK_1_-FE were challenged with VK_1_ injection. An assessment standard for anaphylaxis and anaphylactoid reactions (Table 1) was established with some modifications [14]–[18]. The sums of the scores were determined for each symptom after drug administration.

**Table 1:** An assessment norm for the anaphylactoid reactions and anaphylaxis in dogs.

Grades	Reactions	Scores
0	Normal	0
I	Nose, head, or ear scratching (within 3 min); sneezing, coughing; skin rubeosis	1
II	Nose, head, or ear scratching (greater than 3 min); skin rubeosis; drooling	2
III	Skin rash, vomiting, diarrhea, mania, gait disturbance, unsteadiness of gait	3
IV	Pawing the ground, tumbling, mind sluggishness, somnolence, hypodynamia, wheezing	4
V	Gatism, hematemesis, hemafecia, breathlessness	5
VI	Doom	6

Each symptom in the dogs received a score corresponding to that symptom.

### Blood pressure

The dogs were anesthetized by the intravenous injection of pentobarbital sodium (30 mg/kg) and immobilized on the operating table. The femoral artery was intubated, and the blood pressure was recorded in real time via a BL-420 biological function system.

### ELISA

Before and 10 min after drug administration, blood samples were harvested in a tube containing heparin and centrifuged for 15 min at 2–8°C. The plasma was collected and stored at −20°C. Plasma histamine and IgE concentrations were quantified according to the manufacturers' instructions (Thermo Electron Corporation, USA).

### Spectrophotometry and fluorospectrophotometry

The release of β-hexosaminidase was measured by a fluorospectrophotometric assay. RBL-2H3 mast cells were treated with different concentrations of drugs for 30 min. Aliquots (50 µl) of the supernatants were collected and incubated with 50 µl of 1 mM p-nitrophenyl-N-acetyl-β-d-glucosaminide in 0.1 M sodium citrate (pH 4.5) at 37°C for 2 h. At the end of incubation, 250 µl of carbonate buffer containing 0.1 M Na_2_CO_3_ and 0.1 M NaHCO_3_ (pH 10) was added, and the absorbance resulting from the formation of p-nitrophenol was measured at 405 nm [19], [20]. PBS was used as a blank, and the supernatant from cells stimulated with Triton was considered as the total β-hexosaminidase release. The β-hexosaminidase release rates were calculated as percentages relative to the β-hexosaminidase release from the control.
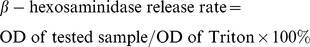



Histamine release was measured by a fluorospectrophotometry assay [19], with some modifications. The supernatant (100 µl) from RBL-2H3 mast cells was added to 40 µl of 0.5 M NaOH and 20 µl of 2.5 mg/ml o-phthalaldehyde and incubated for 30 min. The reaction was terminated by the addition of 10 µl of 3 M HCl. The fluorescence intensity (FI) was measured at an excitation wavelength of 365 nm and an emission wavelength of 465 nm. PBS was used as a blank, and the supernatant from cells stimulated with Triton was considered to represent total histamine release. The histamine release rates were calculated as percentages relative to the histamine release from the control:
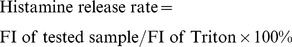



### Fluorescence microscopy observations

According to the instructions provided with the Annexin V-FITC apoptosis detection kit, RBL-2H3 cells were treated with different concentrations of drugs for 30 min, washed twice with cold PBS, and resuspended in binding buffer containing Annexin V-FITC (5 µl) and propidium iodide (5 µl). After 10 min in the dark, the cells were examined and photographed at 400× magnification on an Olympus fluorescent microscope (Eastman Kodak, Rochester, NY, USA).

### Flow cytometry

RBL-2H3 cells were harvested and treated as described above. After 10 min in the dark, the cells were analyzed with a flow cytometer (Guava easyCyte HT, Millipore Co., Hayward, USA).

### Statistical analysis

All statistical analyses were performed using SPSS (Version 18.0). All data were represented as the mean ± SE, and p value <0.05 was considered statistically significant. Data normality was assessed using the Shapiro-Wilk test. Then ANOVA with least significant difference (LSD) were used to analyze quantitative data, including the differences of the levels of histamine, IgE, β-hexosaminidase, the blood pressure, and the percentage of apoptotic cells among the groups. The Kruskal-Wallis test was used to analyze ordinal data, including the change of dog behaviors.

## Results

### Anaphylactoid reactions

#### Behavioristics

The behaviors of the dogs were observed and recorded within 30 min after the first intravenous administration of drugs. Table 2 shows that the dogs in the control and VK_1_-fat emulsion (VK_1_-FE) groups did not display any abnormal behavior. In the Tween-80 group, one dog displayed anaphylactoid grade III symptoms, including scratching his nose or head, ear swinging, skin rash, and gait disturbance; the rest of the dogs displayed grade IV symptoms of pawing at the ground, tumbling, sluggishness, somnolence, or hypodynamia. The average grade and anaphylactoid score were 3.8±0.2 and 13.5±1.1, respectively (*P*<0.01 vs. control). In the 0.25 mg/kg VK_1_ injection group, four dogs displayed anaphylactoid grade IV symptoms, while the other two dogs displayed the grade V symptoms of gatism, hematemesis, hematochezia or breathlessness. Adrenaline hydrochloride injection 0.25 mg and diphenhydramine hydrochloride injection 10 mg were performed intramuscularly to treat these signs. The average grade and anaphylactoid score were 4.3±0.2 and 23.2±4.9, respectively (*P*<0.01 vs. control). In the 0.085 mg/kg VK_1_ injection group, all of the dogs displayed anaphylactoid grade IV symptoms, and the anaphylactoid score was 17.0±1.0 (*P*<0.01 vs. control).

**Table 2:** Grades and scores for anaphylactoid reactions in dogs after the first intravenous administration.

Groups	Doses (mg/kg)	The number of dogs in different grades							Grades	Scores
		0	I	II	III	IV	V	VI		
Control	-	6	0	0	0	0	0	0	0.0±0.0	0.0±0.0
Ovalbumin	2.5	6	0	0	0	0	0	0	0.0±0.0	0.0±0.0
Tween-80	1.0	0	0	0	1	5	0	0	3.8±0.2**	13.5±1.1**
VK_1_-FE	1.0	6	0	0	0	0	0	0	0.0±0.0	0.0±0.0
VK_1_-FE	0.25	6	0	0	0	0	0	0	0.0±0.0	0.0±0.0
VK_1_ injection	0.25	0	0	0	0	4	2	0	4.3±0.2**	23.2±4.9**
VK_1_ injection	0.085	0	0	0	0	6	0	0	4.0±0.0**	17.0±1.0**

Drugs were intravenously administered to the dogs via a micro-injection pump at 0.4 ml/min. Symptoms were observed and recorded for 30 min. The sums of the scores were determined for each symptom. The values are shown as the mean ± SE, n = 6.

**P<0.01 vs. control.

VK_1_: vitamin K_1_; VK_1_-FE: vitamin K_1_-fat emulsion.

#### Plasma histamine concentrations

The standard curve and regression equation for the plasma histamine concentrations were established: y = −0.0032 x^2^+0.1775 x−0.0571, R = 0.9996. Table 3 shows that the plasma histamine concentration in the 0.25 mg/kg VK_1_ injection group increased from 6.41±0.24 before administration to 7.51±0.27 after administration (*P*<0.05). However, compared to the levels before administration, the histamine concentrations in the other groups did not significantly change after administration.

**Table 3:** The change in plasma histamine concentrations after the first intravenous administration.

Groups	Doses (mg/kg)	Histamine concentration (µg/L)	
		Before administration	After administration
Control	-	5.48±0.16	5.90±0.41
Ovalbumin	2.5	6.39±0.55	6.56±0.18
Tween-80	1.0	4.77±0.28	4.89±0.55
VK_1_-FE	1.0	6.94±0.26	7.07±0.22
VK_1_-FE	0.25	4.74±0.15	4.82±0.46
VK_1_ injection	0.25	6.41±0.24	7.51±0.27*
VK_1_ injection	0.085	6.85±0.39	6.86±0.16

Blood samples were taken before and 10 min after drug administration. The plasma histamine concentrations were quantified by ELISA. The values are shown as the mean ± SE, n = 6.

*P<0.05 vs. control.

VK_1_: vitamin K_1_; VK_1_-FE: vitamin K_1_-fat emulsion.

#### Changes in blood pressure

**Figure 1:**
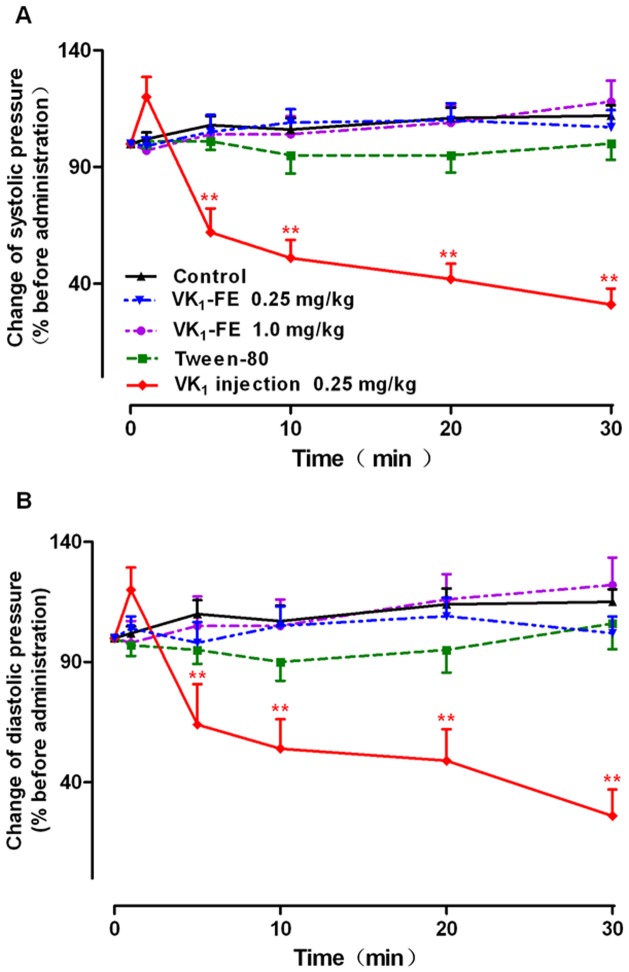
Effect of vitamin K_1_ (VK_1_) injection on systolic blood pressure (A) and diastolic blood pressure (B) in Beagle dogs. The change in blood pressure is presented as the decrease in blood pressure from before to after drug administration. The values are shown as the mean ± SE, n = 6. ^*^ *P*<0.05, ^**^ *P*<0.01 vs. control. VK_1_-FE: vitamin K_1_-fat emulsion.

Figure 1 shows both the systolic (Figure 1A) and diastolic (Figure 1B) blood pressure changes, which are presented as decreases in blood pressure from before to after drug administration. VK_1_ injection clearly affected the blood pressure. The systolic and diastolic blood pressures of dogs administered VK_1_ decreased to 62±4.2% (*P*<0.01) and 64±6.8% (*P*<0.01) at 5 min, respectively. Then, the blood pressure continued to decrease further. At 30 min, the systolic and diastolic blood pressures decreased to 17±2.9% (*P*<0.01) and 26±4.3% (*P*<0.01), respectively. In the control, VK_1_-FE (0.25 and 1.0 mg/kg) and Tween-80 groups, the blood pressure did not obviously change after drug administration when compared with before administration.

### Anaphylaxis and cross-challenge

#### Behavioristics

The dogs were challenged with drugs on the 10th day after the last sensitization. The dogs in the control and VK_1_-FE (0.25 and 1.0 mg/kg) groups did not display any abnormal behavior (Table 4). As predicted, the dogs challenged with ovalbumin displayed obvious abnormalities in the digestive and nervous system behaviors, such as diarrhea, vomiting, tumbling, sluggishness or somnolence, and skin rash. The average grade and anaphylaxis score were 4.0±0.0 (*P*<0.01) and 10.0±1.7 (*P*<0.01), respectively (Table 4). The dogs that were both sensitized and challenged with VK_1_ injection displayed the same symptoms as those in the anaphylactoid experiments. The scores after sensitization three times with 0.25 mg/kg VK_1_ injection were 23.2±4.9, 16.8±1.5, and 15.4±2.1 (Table 5). The ordered decrease in the scores clearly implied that the dogs developed a tolerance to VK_1_ injection. Unfortunately, one dog was very sensitive to VK_1_ injection and presented with skin lesion and gastrointestinal signs such as skin rash, nodule, tumor, vomiting, diarrhea, gait disturbance and unsteadiness of gait. Adrenaline hydrochloride 0.25 mg and diphenhydramine hydrochloride 10 mg were intramuscularly injected to treat these signs. Although some signs faded, the dog remained ill and exhibited frailty and weight loss over the following days. Unfortunately, treatment proved ineffective, and the dog died during the night of the fifth day after the challenge. Although death did not occur within 30 minutes of administration, we believe that it should be included in the list of adverse reactions. The rest of the dogs displayed grade IV symptoms after VK_1_ challenge. The score after challenge with 0.25 mg/kg VK_1_ injection was 14.0±1.6. To distinguish whether the reaction was the results of anaphylaxis or an anaphylactoid reaction, a cross-challenge was performed. When the dogs sensitized with VK_1_ injection were challenged with VK_1_-FE, no abnormal behavior was observed. However, when dogs sensitized with VK_1_-FE were administered VK_1_ injection, severe grade IV symptoms were observed, with a score of 14.2±2.4 (*P*<0.01) (Table 6).

**Table 4:** The grades and scores of symptoms in dogs after the challenge with vitamin K_1_ (VK_1_) injection and vitamin K_1_-fat emulsion (VK_1_-FE).

Groups	Doses (mg/kg)	The number of dogs in different grade					Grades	Scores
		0	I∼III	IV	V	VI		
Control	-	6	0	0	0	0	0.0±0.0	0.0±0.0
Ovalbumin	2.5	0	0	6	0	0	4.0±0.0**	10.0±1.7**
VK_1_-FE	0.5	6	0	0	0	0	0.0±0.0	0.0±0.0
VK_1_-FE	2.0	6	0	0	0	0	0.0±0.0	0.0±0.0
VK_1_ injection	0.25	0	0	5	0	1	4.3±0.3**	14.0±1.6**
VK_1_ injection	0.085	0	0	6	0	0	4.0±0.0**	14.7±1.1**

The dogs were intravenously stimulated with double doses of drug via a micro-injection pump at 0.4 ml/min, and symptoms were then observed and recorded for 30 min. The sum of the scores was determined for each symptom. The values are shown as the mean ± SE, n = 6.

**P<0.01 vs. control.

**Table 5:** The scores of the symptoms shown by the dogs after three sensitizations with vitamin K_1_ (VK_1_) injection and vitamin K_1_-fat emulsion (VK_1_-FE).

Groups	Doses (mg/kg)	Sensitization		
		First	Second	Third
Control	-	0.0±0.0	0.0±0.0	0.0±0.0
Ovalbumin	2.5	0.0±0.0	0.0±0.0	0.0±0.0
VK_1_-FE	1.0	0.0±0.0	0.0±0.0	0.0±0.0
VK_1_-FE	0.25	0.0±0.0	0.0±0.0	0.0±0.0
VK_1_ injection	0.25	23.2±4.9**	16.8±1.5**	15.4±2.1**
VK_1_ injection	0.085	17.0±1.0**	15.5±1.6**	11.0±2.6**

The dogs were sensitized by intravenous drugs every other day for a total of three administrations via a micro-injection pump at 0.4 ml/min; symptoms were then observed and recorded for 30 min. The sum of the scores was determined for each symptom. The values are shown as the mean ± SE, n = 6.

**P<0.01 vs. control.

**Table 6:** Grades and scores for the symptoms presented by the dogs in a cross-challenge experiment.

Sensitization		Stimulation		Grades	Scores
Drugs	Doses (mg/kg)	Drugs	Doses (mg/kg)		
VK_1_	0.25	VK_1_-FE	0.5	0.0±0.0	0.0±0.0
VK_1_-FE	0.25	VK_1_	0.25	4.0±0.0**	14.2±2.4**

Dogs sensitized with 0.25 mg/kg vitamin K_1_ (VK_1_) injection were challenged with 0.5 mg/kg vitamin K1-fat emulsion (VK_1_-FE), and dogs sensitized with 0.25 mg/kg VK_1_-FE were challenged with 0.25 mg/kg VK_1_ injection. Symptoms were observed and recorded for 30 min. The sum of the scores was determined for each symptom. The values are shown as the mean ± SE, n = 6.

**P<0.01 vs. control.

#### Plasma IgE concentrations

A standard curve and regression equation for the plasma IgE concentration were established: y = 3.4×10^−5^ x^2^+0.0182 x−0.0532, R = 0.9998. The plasma IgE concentration in the dogs increased from 48.6±3.0 to 62.7±4.1 (*P*<0.05) after challenge with ovalbumin (Table 7). However, no significant change in the plasma IgE concentrations was observed in the VK_1_ injection and VK_1_-FE groups.

**Table 7:** The change in plasma IgE concentrations after the challenge with vitamin K_1_ (VK_1_) injection and vitamin K_1_-fat emulsion (VK_1_-FE).

Groups	Doses (mg/kg)	IgE concentration (µg/ml)	
		Before sensitization	After stimulation
Control	-	46.9±0.6	48.9±2.8
Ovalbumin	2.5	48.6±3.0	62.7±4.1*
VK_1_-FE	0.5	67.4±4.5	76.5±2.9
VK_1_ -FE	2.0	51.1±1.0	53.0±6.3
VK_1_ injection	0.25	56.1±3.0	53.1±3.1
VK_1_ injection	0.085	45.5±1.6	45.5±4.2

Blood samples were collected into a tube containing heparin at 10 min after drug administration. The plasma IgE concentrations were determined by ELISA. The values are shown as the mean ± SE, n = 6.

*P<0.05 vs. control.

### Effect on degranulation in RBL-2H3 cells

#### β-hexosaminidase release

**Figure 2:**
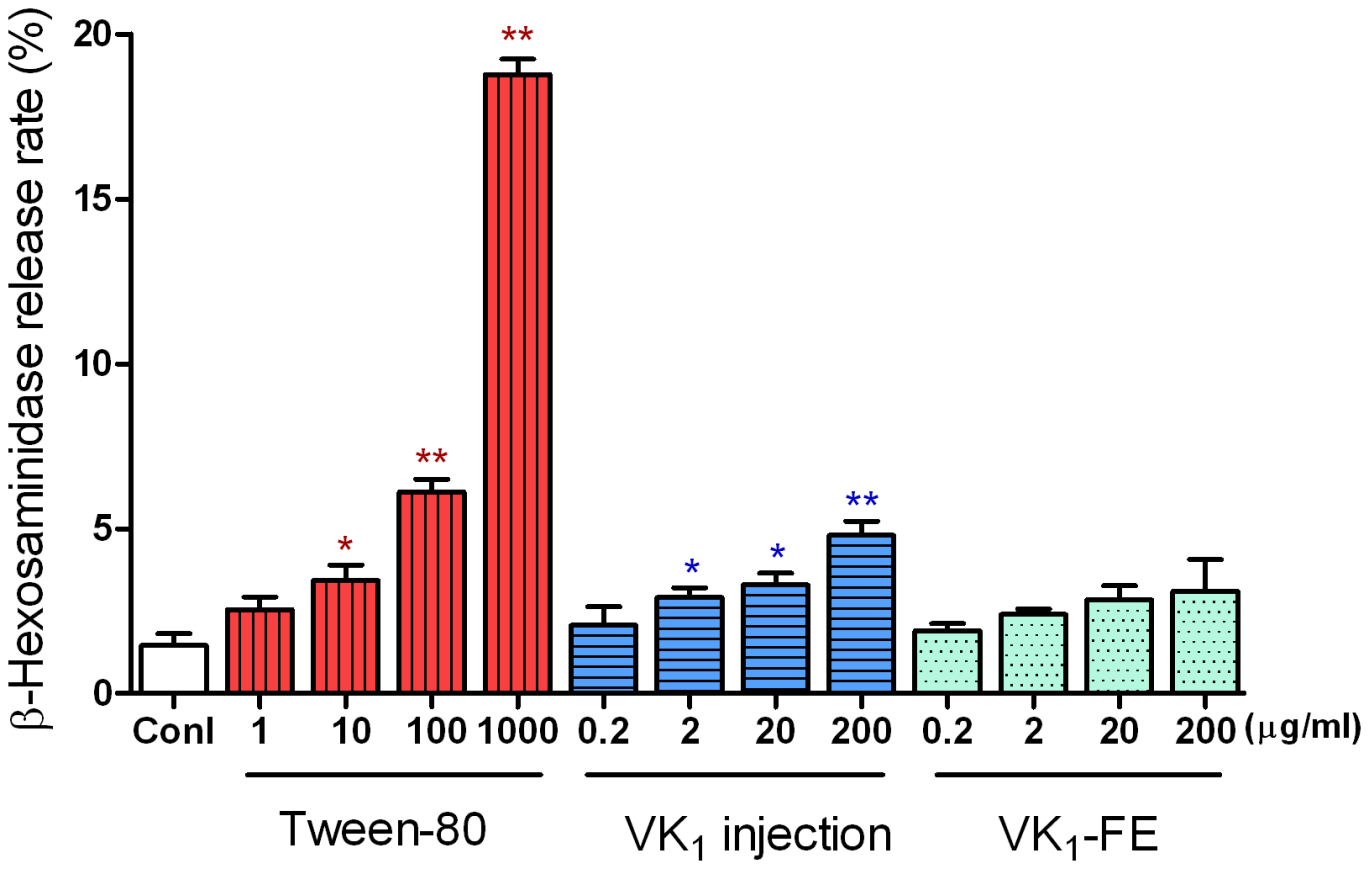
Effect of vitamin K_1_ (VK_1_) injection on β-hexosaminidase release in RBL-2H3 cells. RBL-2H3 cells were treated with the different drugs for 30 min. The supernatants were collected, and the absorbance was measured at 405 nm. The supernatant from cells stimulated with Triton was considered to represent the total β-hexosaminidase release. The β-hexosaminidase release rates are shown as the mean ± SE, n = 6. ^*^ *P*<0.05, ^**^ *P*<0.01 vs. control (conl). VK_1_-FE: vitamin K_1_-fat emulsion.

Figure 2 shows that VK_1_ injection treatment directly stimulated RBL-2H3 cells to release β-hexosaminidase. Treatment with 2, 20, or 200 µg/ml VK_1_ injection induced a significant increase in β-hexosaminidase release to 2.9±0.3%, 3.2±0.4%, and 4.8±0.4%, respectively (*P*<0.05 or *P*<0.01), when compared with the release from the control (1.5±0.3%). Tween-80 (10–1000 µg/ml) also induced β-hexosaminidase release from 3.4±0.4% to 18.8±0.4%. In contrast, no significant difference in the β-hexosaminidase release rate was observed between the VK_1_-FE group and the control group.

#### Histamine release

**Figure 3:**
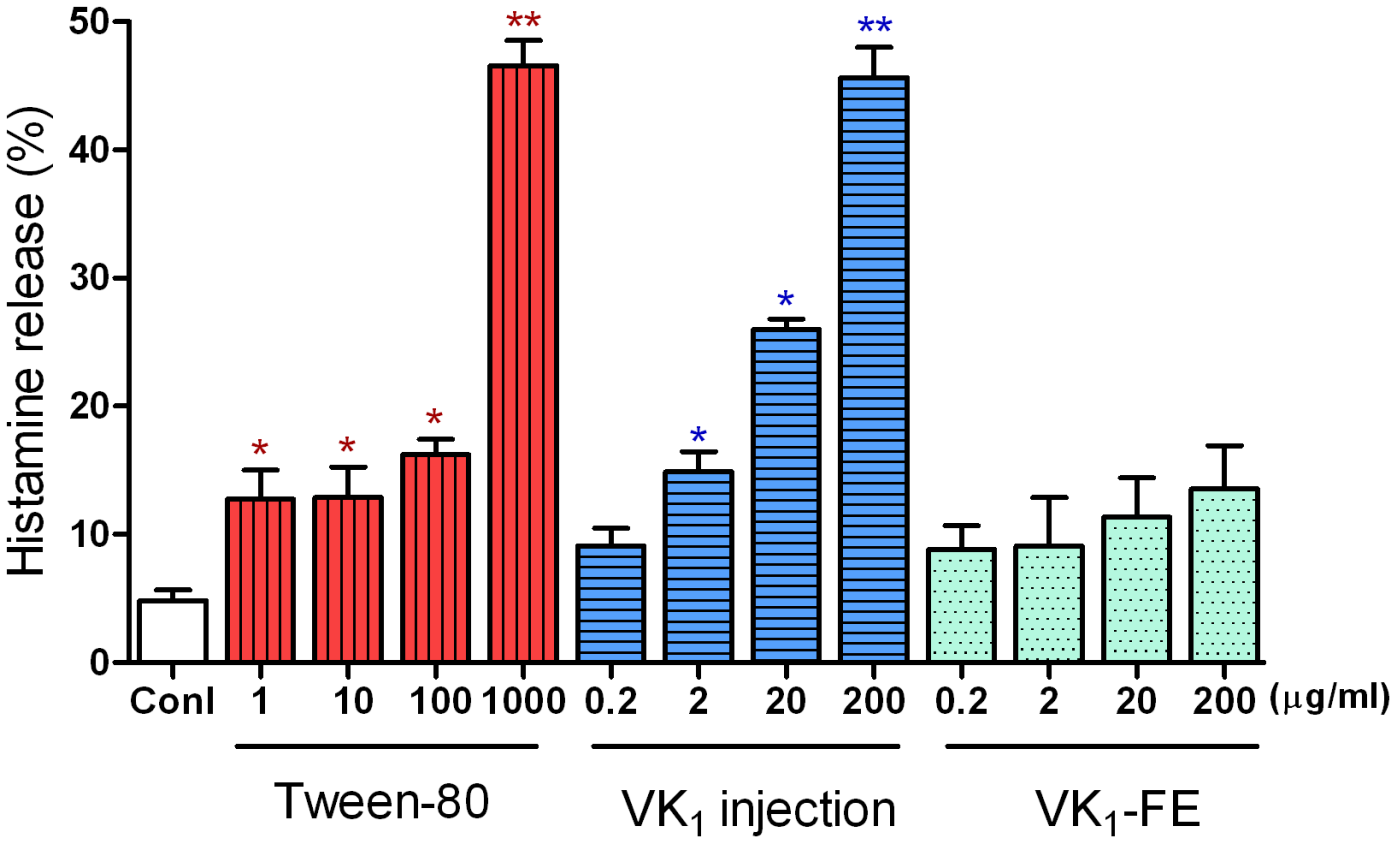
Effect of vitamin K_1_ (VK_1_) injection on histamine release in RBL-2H3 cells. RBL-2H3 cells were treated with the different drugs for 30 min. The supernatants were collected and measured by spectrofluorometry. PBS was used as a blank, and the supernatant from cells stimulated with Triton was considered to represent the total histamine release. The histamine release rates are shown as the mean ± SE, n = 6. ^*^ *P*<0.05, ^**^ *P*<0.01 vs. control (conl). VK_1_-FE: vitamin K_1_-fat emulsion.

The effect of VK_1_ on histamine release from non-antigen-stimulated RBL-2H3 cells was evaluated. Figure 3 shows that the histamine release in the treatment groups treated with 2, 20, or 200 µg/ml VK_1_ injection was 14.8±1.6%, 26.0±0.8%, and 45.7±2.4%, respectively, indicating that VK_1_ injection increased histamine release in a concentration-dependent manner. Similarly, Tween-80 (1–1000 µg/ml) increased the histamine release from 12.7±2.2% to 46.6±2.0% when compared with the release by control cells (4.9±0.8%)(*P*<0.05 or *P*<0.01). As expected, and in agreement with the previous results, VK_1_-FE did not induce histamine release.

#### Apoptosis of RBL-2H3 cells

**Figure 4:**
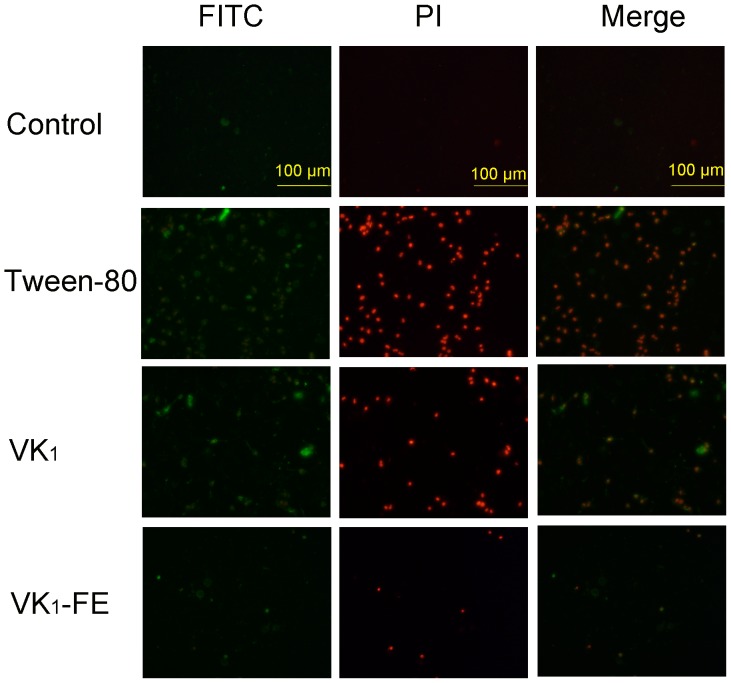
Effect of vitamin K_1_ (VK_1_) on apoptosis of RBL-2H3 cells by fluorescence staining. AnnexinV-FITC-stained cells appear green at the early stage of apoptosis. PI caused the cells to appear red during the middle to late stages of apoptosis. The magnification is 400×. The merged green and red images represent total apoptosis. VK_1_-FE: vitamin K_1_-fat emulsion.

The morphology of apoptotic cells was assessed by fluorescence microscopy using the Annexin V-FITC and propidium iodide staining method. During the early stages of apoptosis, the membrane phospholipid phosphatidylserine translocates from the inner to outer leaflet of the cytomembrane. Annexin V-FITC binds to cells with exposed phosphatidylserine, causing the cells to appear green. During the middle-late stages of apoptosis, the integrity of the cytomembrane is disrupted, and propidium iodide (a reactive dye) can pass through the cytomembrane and embed in the DNA, causing the cells to appear red. Thus, normal cells are colorless, early apoptotic cells are bright green, and middle to late apoptotic cells are yellow (representing equal amounts of red and green). The results indicated that both VK_1_ injection and Tween-80 significantly induced the apoptosis of RBL-2H3 cells compared with the control (Figure 4). In contrast, VK_1_-FE treatment caused only a spot of yellow, and no significant apoptosis was detected compared with the control (Figure 4).

**Figure 5:**
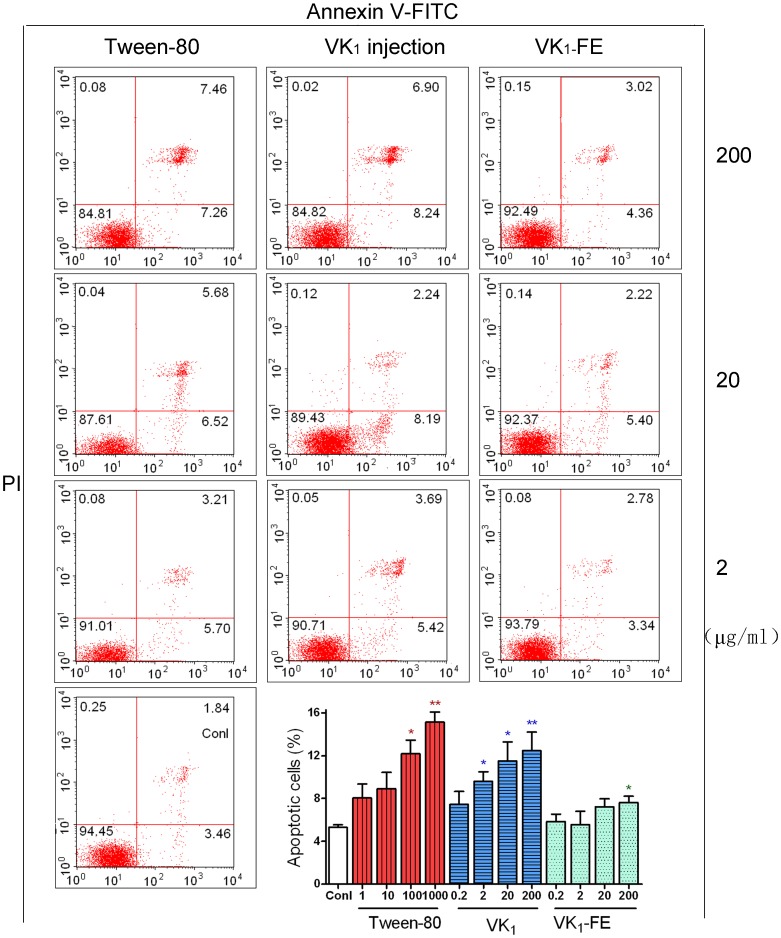
Effect of vitamin K_1_ (VK_1_) injection on the apoptosis of RBL-2H3 cells by flow cytometry. Apoptotic cells were identified by Annexin V-FITC and PI staining. The concentrations of Tween-80 are 1000, 100, and 10 µg/mL, from top to bottom. The values are shown as the mean ± SE, n = 6. ^*^ *P*<0.05, ^**^ *P*<0.01 vs. control (conl). VK_1_-FE: vitamin K_1_-fat emulsion.

Flowcytometry was used to explore the concentration-response relationship between VK_1_injection treatment and apoptosis. Annexin V-FITC/PI staining indicated that both VK_1_ injection and Tween-80 induced apoptosis in RBL-2H3 cells in a concentration-dependent manner. Figure 5 showed that the percentage of apoptotic cells in the control was 5.3±0.6%. The percentage of apoptotic cells in the Tween-80 (1–1000 µg/ml) group clearly increased from 8.0±1.8% to 15.1±2.0% (*P*<0.01). The percentage of apoptotic cells in the groups treated with 2, 20, and 200 µg/ml VK_1_ injection increased significantly to 8.2±1.9%, 9.1±1.4%, and 14.7±3.4%, respectively (*P*<0.05 or *P*<0.01),when compared with the control. However, VK_1_-FE did not induce apoptosis at concentrations of 0.2–20 µg/ml, although apoptosis was observed at 200 µg/ml.

## Discussion

Anaphylaxis is most often triggered by repeated exposure to allergens such as drugs, which can stimulate the body to produce antibodies through an IgE-mediated immune response. Meanwhile, drugs can cause anaphylactoid reaction which displays the same clinical manifestations as anaphylaxis. However, a different mechanism exists. Anaphylactoid reactions are non-IgE-mediated and do not require a history of exposure. In both IgE- and non-IgE-mediated reactions, mast cells and basophils rapidly release histamine, β-hexosaminidase and tryptase via different trigger mechanisms [19], [21]. Because anaphylaxis and anaphylactoid reactions are clinically indistinguishable, reports of anaphylactoid reactions and anaphylaxis are confused. The nature of the adverse reaction to VK_1_ injection is one example of this uncertainty.

The anaphylaxis and anaphylactoid reactions induced by drugs are related to hereditary capacity, immune status, the drug delivery route, the identity of the drug, and metabolism, among other factors. Completely simulating these responses *in vitro* is difficult. Therefore, selecting and establishing the appropriate animal model is very important. Compared with other experimental animals, dogs and humans are more likely to experience similar symptoms [22], [23], and dogs are more sensitive to anaphylactoid reactions than any other animals. The degranulation of basophils or mast cells is an important element in the study of anaphylaxis and anaphylactoid reactions. RBL-2H3, a continuous rat cell line that is useful for *in vitro* studies [24], has been used extensively to study signaling pathways involved in degranulation [25] and IgE-FcεRI interactions.

Behavioristics is considered to be a vital, simple and intuitive method to determine the type of adverse reaction. The present study showed that dogs experienced serious multiorgan symptoms, increases in plasma histamine concentrations, and sharp decreases in blood pressure after the first intravenous VK_1_ injection. Undoubtedly, the results demonstrate that VK_1_ injection induces an anaphylactoid reaction.

VK_1_ is a lipid-soluble substance. For preparation as an injection, the use of a solubilizer such as Tween-80 is required. When 1 mg/kg Tween-80 was administered to the dogs, anaphylactoid reactions appeared; these results are in agreement with previous reports [12], [14], [26]. Other drugs that require solubilizers containing Tween-80, such as qingkailing injection, shengmai injection, the anti-neoplastic agent paclitaxel and the immunosuppressant cyclosporine, also cause obvious anaphylactoid reactions [18], [27], [28]. However, it is the low dose of Tween-80 (1 mg/kg) that did not lead to the significant changes in the plasma histamine concentration and blood pressure. The plasma histamine levels sharply increased in the dogs upon the administration of 25 mg/kg Tween-80 [13].

VK_1_-FE is a preparation in which VK_1_ is dissolved in lecithin and is a steady O/W emulsion without any solubilizer. This preparation can be used to help explain the effect of the solubilizer on adverse reactions. Abnormal behaviors were not observed in dogs administered VK_1_-FE without Tween-80. Furthermore, no significant change in plasma histamine was observed in these dogs. The results revealed that VK_1_-FE does not induce an anaphylactoid reaction. Therefore, VK_1_ is not the trigger that initiates the anaphylactoid reaction in response to VK_1_ injection.

Upon challenge with VK_1_, the dogs showed the same abnormal multisystem symptoms as dogs that had been sensitized with VK_1_ injection. Determining whether the multisystem symptoms were indicative of anaphylaxis was difficult. Therefore, we devised a cross-challenge experiment. The results showed that abnormal behaviors were not observed in dogs sensitized with VK_1_ injection that were later challenged with VK_1_-FE. In dogs sensitized with VK_1_-FE that were challenged with VK_1_ injection, severe abnormal behaviors were observed. Furthermore, no significant change in plasma IgE concentrations was observed in the VK_1_ injection and VK_1_-FE groups. The results indicated that the abnormal behaviors of the dogs administered VK_1_ injection in the anaphylaxis experiment (during both sensitization and challenge) were due to anaphylactoid reactions, not anaphylaxis.

We further studied the mechanisms underlying the anaphylactoid reactions. It is well-known that measurements of histamine and β-hexosaminidase release are common methods for detecting the activation of RBL-2H3 cells stimulated *in vitro*. In the present study, VK_1_ injection treatment directly resulted in the degranulation of the non-antigen-sensitized RBL-2H3 cells in a concentration-dependent manner. These results demonstrated that the anaphylactoid reaction resulting from VK_1_ injection treatment was due to the release of histamine, β-hexosaminidase, and other inflammatory factors through a non-IgE-mediated pathway, and these factors are known to cause pruritus, acute inflammation, and vasodilatation. Simultaneously, the percentages of both early and late-stage apoptotic cells markedly increased in the VK_1_ injection treatment group. The results demonstrate that the release of histamine and other inflammatory factors induced by VK_1_ injection is the result of phosphatidylserine exposure and an increase in cell membrane permeability in RBL-2H3 cells. VK_1_-FE did not induce apoptosis in RBL-2H3 cells, suggesting that apoptosis is not induced by VK_1_. However, RBL-2H3 cells treated with Tween-80 displayed severe apoptosis in a concentration-dependent manner, suggesting that the apoptosis induced by VK_1_ injection may be due to the presence of Tween-80 in the formulation.

In conclusion, VK_1_ injection induces anaphylactoid reactions, not anaphylaxis. However, VK_1_ itself is not involved in the anaphylactoid reaction. The trigger may be the solubilizer. This conclusion provides a basis for generating VK_1_ preparations without severe adverse reactions. Altering the VK_1_ preparation, decreasing the solubilizer dosage, or using a highly safe solubilizer may be good strategies to reduce or eradicate the anaphylactoid reactions caused by treatment with VK_1_ injection. Properly discerning the category and pathogenesis of adverse drug reactions will aid our ability to prevent and reduce adverse reactions to VK_1_ preparations.

## References

[1] Vandermeir J (1968) Vitamin K. Thromb Diath Haemorrh 29: 1–95.

[2] Fiore LD, Scola MA, Cantillon CE, Brophy MT (2001) Anaphylactoid reactions to vitamin K. J Thromb Thrombolysis 11: 175–183. 11406734 10.1023/a:1011237019082

[3] Pereira SP, Williams R (1998) Adverse events associated with vitamin K1: results of a worldwide postmarketing surveillance programme. Pharmacoepidemiol Drug Saf 7: 173–182. 15073995 10.1002/(SICI)1099-1557(199805/06)7:3<173::AID-PDS343>3.0.CO;2-8

[4] SFDA PRC (2011) Alert regarding serious allergic reactions to vitamin K1 injection. National Center for ADR Monitoring

[5] Sousa T, Hunter L, Petitt M, Wilkerson MG (2010) Letter: Localized cutaneous reaction to intramuscular vitamin K in a patient with acute fatty liver of pregnancy. Dermatol Online J 16: 16. 21199642

[6] Sommer S, Wilkinson SM, Peckham D, Wilson C (2002) Type IV hypersensitivity to vitamin K. Contact Dermatitis 46: 94–96. 11918602 10.1034/j.1600-0536.2002.460206.x

[7] Riegert-Johnson DL, Volcheck GW (2002) The incidence of anaphylaxis following intravenous phytonadione (vitamin K1): a 5-year retrospective review. Ann Allergy Asthma Immunol 89: 400–406. 12392385 10.1016/S1081-1206(10)62042-X

[8] Wilkins K, DeKoven J, Assaad D (2000) Cutaneous reactions associated with vitamin K1. J Cutan Med Surg 4: 164–168. 11003724

[9] Yang G-H, Lei Z-B (2009) The analysis of 45 cases of the anaphylactic shock indused by vitamin K1 injection. Chin J of Clinical Rational Drug Use 2.

[10] Martin JC (1991) Anaphylactoid reactions and vitamin K. Med J Aust 155: 851. 10.5694/j.1326-5377.1991.tb94070.x 1745193

[11] Riegert-Johnson DL, Kumar S, Volcheck GW (2001) A patient with anaphylactoid hypersensitivity to intravenous cyclosporine and subcutaneous phytonadione (vitamin K1). Bone Marrow Transplant 28: 1176–1177. 11803365 10.1038/sj.bmt.1703305

[12] Coors EA, Seybold H, Merk HF, Mahler V (2005) Polysorbate 80 in medical products and nonimmunologic anaphylactoid reactions. Ann Allergy Asthma Immunol 95: 593–599. 16400901 10.1016/S1081-1206(10)61024-1

[13] Qiu S, Liu Z, Hou L, Li Y, Wang J, et al (2013) Complement activation associated with polysorbate 80 in beagle dogs. Int Immunopharmacol 15: 144–149. 23159336 10.1016/j.intimp.2012.10.021

[14] Sun WW, Li YK, Zhang JY (2011) [Anaphylactoid reactions inducing effect of polysorbate 80 and polysorbate 80 contained Houttuynia cordata injection on beagle]. Zhongguo Zhong Xi Yi Jie He Za Zhi 31: 90–93. 21434352

[15] Wang Z, Wang D, Sui Y, Cui H, Yu Y (2012) Experimental study on anaphylaxis of qingkailing injection and its components on Beagle dogs. J Tradit Chin Med 32: 641–645. 23427403 10.1016/s0254-6272(13)60085-0

[16] Wang Z, Wang D, Yu Y, Li Y, Sui Y, et al (2011) Experimental model of histamine-induced anaphylactoid reaction on beagle dogs. Zhongguo Zhong Yao Za Zhi 36: 1842–1844. 22016944

[17] Liang A, Li C, Hao R, Cao C, Yi Y, et al (2010) [Pseudoanaphylactoid reaction analysis of Chinese herbal injections in Beagle dogs]. Zhongguo Zhong Yao Za Zhi 35: 2328–2333. 21137349

[18] He P, Li F, Tang R, Li Y, Hao W, et al (2012) [Experimental study on anaphylactoid reactions induced by different components of shengmai injection (new production process) on Beagle dogs]. Zhongguo Zhong Yao Za Zhi 37: 1880–1884. 23019862

[19] Vo T, Kong C, Kim S (2011) Inhibitory effects of chitooligosaccharides on degranulation and cytokine generation in rat basophilic leukemia RBL-2H3 cells. CARBOHYDRATE POLYMERS 84: 649–655.

[20] Na HJ, Moon PD, Lee HJ, Kim HR, Chae HJ, et al (2005) Regulatory effect of atopic allergic reaction by Carpopeltis affinis. J Ethnopharmacol 101: 43–48. 15893895 10.1016/j.jep.2005.03.026

[21] Nishikawa H, Kitani S (2011) Gangliosides inhibit bee venom melittin cytotoxicity but not phospholipase A(2)-induced degranulation in mast cells. Toxicol Appl Pharmacol 252: 228–236. 21334356 10.1016/j.taap.2011.02.011

[22] Ng W, Lobach AR, Zhu X, Chen X, Liu F, et al (2012) Animal models of idiosyncratic drug reactions. Adv Pharmacol 63: 81–135. 22776640 10.1016/B978-0-12-398339-8.00003-3

[23] Shenton JM, Chen J, Uetrecht JP (2004) Animal models of idiosyncratic drug reactions. Chem Biol Interact 150: 53–70. 15522261 10.1016/j.cbi.2004.09.001

[24] Huang FH, Zhang XY, Zhang LY, Li Q, Ni B, et al (2010) Mast cell degranulation induced by chlorogenic acid. Acta Pharmacol Sin 31: 849–854. 20581858 10.1038/aps.2010.63 PMC4007731

[25] Funaba M, Ikeda T, Abe M (2003) Degranulation in RBL-2H3 cells: regulation by calmodulin pathway. Cell Biol Int 27: 879–885. 14499669 10.1016/s1065-6995(03)00177-x

[26] Sun W, Li Y, Wang N, Du F, Hao W, et al (2011) [Anaphylactoid reactions induced by polysorbate 80 on Beagle dogs]. Zhongguo Zhong Yao Za Zhi 36: 1874–1878. 22016951

[27] Wang Z, Wang D, Yu Y, Sui Y, Cui H, et al (2011) [Study on allergenicity of chlorogenic acid in Qingkailing injection]. Zhongguo Zhong Yao Za Zhi 36: 1870–1873. 22016950

[28] Weiss RB, Donehower RC, Wiernik PH, Ohnuma T, Gralla RJ, et al (1990) Hypersensitivity reactions from taxol. J Clin Oncol 8: 1263–1268. 1972736 10.1200/JCO.1990.8.7.1263

